# Trajectories of performance change indicate multiple dissociable links between working memory and fluid intelligence

**DOI:** 10.1038/s41539-021-00111-w

**Published:** 2021-11-29

**Authors:** Aaron Cochrane, C. Shawn Green

**Affiliations:** 1 Faculty of Psychology and Education Sciences, University of Geneva, 40 Boulevard du Pont-d’Arve, 1211 Genève, Switzerland; 2 Department of Psychology, University of Wisconsin—Madison, 1202 W Johnson St, Madison, WI 53706 USA

**Keywords:** Human behaviour, Working memory

## Abstract

Many areas of psychology assume that performance on tasks of interest is stable through time. Here, using time-sensitive modeling of working memory task performance, we show not only was this assumption incorrect, but that certain components of the performance trajectory (e.g., final task performance; rate of change) were independently predictive of fluid intelligence. This fact has clear implications for theoretical frameworks linking working memory and fluid intelligence, and beyond.

Many theories in experimental psychology are paradigmatically grounded in inferences derived from participants’ performance aggregated across many trials of one or more tasks. Here we consider one well-known example relating fluid intelligence (Gf), which encompasses the ability to think abstractly and to learn independently of previous experience, with working memory (WM)^1–3^.

Previous work in the domain has utilized methods and analytic approaches that assume not only that the *constructs of interest* (e.g., WM) are reasonably stable through time, but also that *performance on the tasks* indexing the given constructs is likewise stable. Indeed, to our knowledge, all previous work examining the relations between WM and Gf has aggregated participant data over trials (e.g., calculating percent correct or d-prime). In performing such an aggregation-based analysis it is assumed either: (A) that participants’ ability to complete the task remains stationary throughout the task (i.e., that data across trials is independently and identically distributed; *iid*); or (B) that any systematic changes in performance that occur are irrelevant with respect the construct of interest. If these assumptions are not met, then the previously observed links between WM and Gf are, at a minimum, underspecified.

If individuals’ performance on WM tasks changes through time (which as we will see below, it does, even across less than 100 trials of experience), what might be possible reasons for the previously observed links between aggregated WM performance and measures of Gf? One is that Gf shares *processing constraints* with WM^1–3^, leading to correlations between Gf scores and WM performance that are stationary across the measured timescale. A second possibility is related to the idea that Gf reflects “the ability to learn”^4–7^. This might predict that correlations would be observed between Gf scores and the *rate of change* in WM performance. Finally, Gf may relate to the ability to perform novel tasks well from the very first attempt, manifesting as correlations between Gf scores and *initial* measured cognitive task performance^8^. While these possibilities are neither exhaustive nor necessarily independent, each is supported by previous work and theory, and all three are confounded by the aggregation of performance over time (see Fig. 1).

**Fig. 1:**
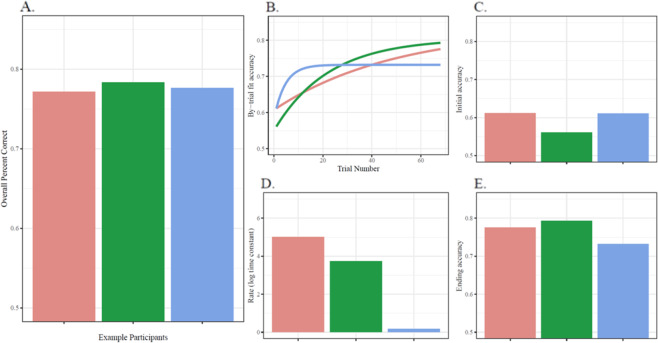
Example data. WM data from three participants were selected specifically to visualize certain problems with typical aggregation-based measures. Panel **A** shows the participants’ similar overall average accuracy. An analysis relating overall WM performance to Gf would expect similar levels of Gf in these participants. Yet, when examining the participants’ trajectories of performance on the WM task (**B**), they evince “same overall performance” in quite different ways. They differ markedly in initial accuracy (**C**), rate of change (**D**), and in final accuracy (**E**). As such, more detailed theories linking WM and Gf would not make identical predictions for the three participants.

Here we tested these potential explanations for links between WM and Gf task performance. Eighty-seven participants completed standard measures of Gf (matrix reasoning) and of WM (digitized Corsi). We then modeled WM task performance as a continuous function of time (via a nonlinear mixed-effects Bayesian regression) in order to assess participants’: Initial Accuracy, Rate of Change, and Final Accuracy^9^; see Supplementary Information for model specification. The extent to which these components were related to matrix reasoning task performance was then assessed.

In examining the links with Gf, initial WM accuracy had no convincing evidence for a relation with Gf (BF_log3_ = 0.18). In contrast, both rate of change on the WM task (*r*(85) = −0.30, CI = [−0.49, −0.08], BF_log3_ = 2.05) and final accuracy (*r*(85) = 0.33, CI = [0.11, 0.52], BF_log3_ = 2.99) showed evidence of relations to Gf (see Fig. 2; the same pattern holds when calculating Spearman ρ). In a confirmation of these effects’ independence, a regression model was run containing main effects for both predictors (rate of change and final accuracy), and each remained reliable when controlling for the other (rate of change *b* = −0.111, CI = [−0.213,−0.008], Δ*R*^2^ = 0.046; final accuracy *b* = 0.428, CI = [0.112,.745], Δ*R*^2^ = 0.073; note that these results remain when using robust regression to minimize the influence of high-leverage datapoints, or when including starting accuracy as a covariate; see Supplementary Note 1).

**Fig. 2:**
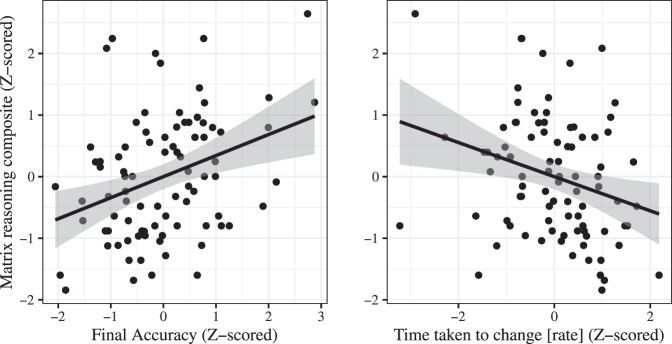
Correlations between Gf and two components of participants’ working memory trajectories. Components were the final accuracy (*r* = 0.33, BF_log3_ = 2.99) and rate of change (*r* = −0.30, BF_log3_ = 2.05). Shaded area indicates 95% CI.

The present results provide new insight into the mechanistic bases of previously observed correlations between WM and Gf. The relationship between final estimated WM accuracy and Gf supports the prevailing view in the literature that the associations between Gf and WM are strongly rooted in relatively stable processing characteristics that vary between individuals. Additional support for this view is found by the strong relationship between estimated final WM accuracy and overall accuracy (i.e., with the typical measure that would be used in the field; the relation between overall and final WM accuracy: *r*(85) = 0.96, CI = [0.93,0.97], BF_log3_ = 89.19). This statistical near-isomorphism supports the interpretation that, throughout our results, ending accuracy is an index of stable performance and can be interpreted as a proxy for the standard overall-accuracy measure (i.e., that the standard method of analysis is largely capturing the variance that would be expected by theories of working memory, particularly with larger numbers of trials). Yet the present study also provided novel evidence for an independent link between Gf and the rate of change within WM tasks, with change occurring in less time (i.e., steeper trajectories) being associated with higher Gf. This, along with the lack of evidence for a correlation between the rate of change and overall WM accuracy (BF_log3_ = 0.26), further supports the independence of the two links between WM and Gf. Interestingly, there was no reliable support for links between Gf measures and initial WM accuracy in the current data. This has particularly pertinent implications for research batteries with shortened tasks (i.e., shorter WM tasks will tend to load less strongly on the measures of interest; see also Supplementary Note 2, Supplementary Fig. 4, and Supplementary Table 2).

The work here opens new directions in examining WM/Gf links. For example, our experimental design precluded time-sensitive analyses of Gf, and linking the dynamics of WM with those of Gf will be a topic of further study. We conclude by suggesting that the implications of the approach used here, both in terms of theory and in terms of practice, are much broader than the examination of WM and Gf. Indeed, there are many areas of cognitive psychology that have relied overwhelmingly upon aggregation-based statistics. The foundations of major frameworks are thus predicated on the not-closely-tested belief that performance on key tasks is stable through time. If, for instance, correlations across tasks in the literature have been partially driven by dynamics of change, it would necessitate a reinterpretation of those frameworks. Although researchers sometimes attempt to address this problem by removing some trials as “practice trials,” this (A) simply replaces one assumption of stationarity with another (often in a somewhat unprincipled way, as it is unclear how many trials should be removed to ensure that performance in the remaining sample is stationary), and (B) as our results here show, is actually discarding signal, not noise. However, while our results suggest that researchers should avoid discarding trials whenever possible, the use of time-sensitive analyses on pilot data may provide empirical support for timescales of change within a given task and therefore what amount of practice would be appropriate if it were to be implemented (i.e., where most participants have reached reasonably stable performance). To address such issues future work in this domain, and many other areas of experimental psychology, may benefit from a time-sensitive understanding of cognitive task performance.

## Methods

### Overview

Procedures were approved by the UW–Madison Institutional Review Board, and all participants provided informed consent prior to participation. Two types of tasks were completed in a dimly-lit room by each participant (recruited from Introduction to Psychology courses; *n* = 87, *m*_age_ = 19.1 years, sd_age_ = 0.73, 50 female; see Supplementary Fig. 1). Gf scores were calculated by independently z-scoring, and then averaging the percent correct, on two measures of matrix reasoning^10,11^. WM was assessed using 68 trials of a digitized forward spatial span similar to a Corsi-block-tapping task (note that some participants received explicit feedback on this task while others did not; this confirmed the robustness of our results to feedback-related factors). Tasks were presented in an Internet browser using Qualtrics.

### Procedure

The experimenter, who was blind to the hypotheses of the study, initiated the computerized tasks on Dell desktop computers running Windows 7. The experimenter was blind to the presence or absence of feedback to the participant. No other persons were in the room as the participant completed the tasks. All stimuli were presented, using the Qualtrics survey software, in Google Chrome on Dell 22-inch monitors. All instructions were provided in writing via Qualtrics. Tasks were designed to last approximately 45 min total (see Supplementary Fig. 1). The two task types, WM (spatial span) and Gf (two matrix reasoning assessments) were counterbalanced pseudorandomly across participants, and an order effect was included as a covariate in the model of change over time (see Supplementary Table 1).

Within the spatial span task (similar to “Corsi block-tapping” or the recall component of “symmetry span”^12–14^), at the beginning of each trial, a display of 12 empty squares was randomly placed within an invisible 5×5 grid in the center of the screen. After 500 ms, one square would fill with color for .5 s before returning to an empty outline. Immediately a different square would fill with color, and so on until the trial’s set size was reached. A sequence of random non-repeating target locations was defined through these color changes. After the sequence participants used their mouse to reproduce the sequence of targets. In the feedback-present condition, participants were provided with the percent correct they received on each trial; in the feedback-absent condition, they were not given any information about their performance. Set sizes were blocked in groups of four in the following order: four of set size 6, four of set size 8, four of set size 5, and four of set size 7. This series of 16 trials was repeated four times. There were two very easy catch trials of set size 3 in between the first and second blocks and between the third and fourth blocks for a total of 68 trials. There was a break between the second and third blocks (i.e., halfway through the task). Participants were not informed of the set size on any trial. Identical trial orders of set sizes ensured that participants’ experience in the task was comparable and variations in change over time would not be due to variations in histories of trial difficulties.

### Analyses

Chance accuracy on matrix reasoning tasks was defined as failing to be significantly above the guessing rate of 12.5%, utilizing a one-tailed binomial test. This translated to excluding participants who scored 3 or lower on the Sandia matrices^10^ or 4 or lower on the UCMRT^11^. Chance performance (set at a 50% criterion) was also assessed on spatial span catch trials of set size 3, however, no participants were excluded by this criterion.

WM performance was modeled in a nonlinear Bayesian model using the R package *brms*^15^. Fixed effects of task presentation order and the presence of feedback were included (and were confirmed to be not reliable on their own or in interactions with other effects), as well as random effects for each participant’s initial accuracy, rate of change in accuracy (a time constant and inverse of speed), and asymptotic accuracy (see Supplementary Information). From this model, mean parameter estimates of participant-level initial, rate, and final accuracy were extracted and used in further analyses. Correlations used bootstrapped confidence intervals and were reported with the log (base 3) of the Bayes Factor, such that conventional thresholds for evidence in favor of a hypothesis would be ±1.

### Spatial span nonlinear model

See Supplementary Note 3 for the model formula. Data was fully disaggregated when fit (i.e., every mouse click was a separate line in the data set, with accuracy being 0 or 1). Set size was mean-centered (at 6.5) in order to have all other effects estimated at this intermediate value. Starting and asymptotic accuracy parameters were estimated on inverse-logit scales in order to provide unbounded parameter ranges associated with bounded ranges of predicted accuracies. This, in effect, provided parameter-level generalized mixed-effects models accounting for the systematic change in accuracy due to set size differences (i.e., participant-level psychometric functions; see Supplementary Table 1 and Supplementary Figs. 2, 3). Rate parameters were estimated on a binary log scale and included an additive offset of 2 (see Supplementary Note 3) in order to avoid allowing the rate [50%-of-change time constant] to have impossibly small values. This constraint assists in model convergence. Note that, while previous research has addressed putatively separable components of learning (e.g., linear, log-linear, or log−log growth curve models), the fully nonlinear model implemented here prevents the conflation of components of learning inherent in many other forms of learning estimates.

The model was estimated using a Bernoulli objective function (i.e., “family”). All priors were default where possible, and otherwise were minimally informative; we note that the between-participants results of interest were reliant solely on default priors. The model was run for 4 chains, with 5,000 iterations per chain and the first 3,000 iterations per chain discarded as warm-up. As is clear in the model formula, the primary nonlinear formula involves an exponential change in accuracy as a function of trial number. This curve is bounded between 0 and 1 by the parameters’ generalized linear models (i.e., logistic regression for asymptote and start parameters; in addition, the rate parameter was estimated as a time constant on a binary log scale).

After model sampling was completed, point estimates for each parameter were extracted from the model (i.e., means of the distributions of participant-level estimates using the *brms::coef* function). This method paralleled the standard approach in individual-differences studies, in which task-level aggregation (e.g., using mean accuracies or psychometric function thresholds) is followed by cross-task tests of relations.

### Exclusions

Initial recruitment targets were 50 retained participants per feedback condition. The feedback-present group initially included 55 participants; 12 were excluded for chance performance on the UCMRT^2^, 6 were excluded for chance performance on Sandia matrices^3^, and 0 were excluded for chance performance on set-size-3 spatial span. The feedback-absent group initially included 53 participants^8^; were excluded for chance performance on the UCMRT, 1 was excluded for chance performance on the Sandia matrices, and none were excluded for chance performance on set-size-3 spatial span. This left 42 participants in the feedback-present group and 45 participants in the feedback-absent group. Participants were recruited from a college population. While this reduced the heterogeneity of the population (and thus the extent to which the results can be extrapolated), this sampling strategy was preferred given that our primary research questions were to examine the extent to which our novel analysis approach provided new insight into questions that had overwhelmingly been addressed previously in similar populations (and thus, if we had utilized a different population it would have been unclear whether our results spoke to the previous work). Our results indicate that future work in a more heterogenous sample would be beneficial—particularly in low performers (where low rates of change could easily mask what would eventually be high asymptotic performance).

### Power

Power to detect correlations was calculated, and with 87 participants we had 80% power to detect correlations of at least 0.295. Correlations thus had well over 80% power to detect correlations of 0.48, which has been reported as the average correlation between WM and Gf (Ackerman, Beier, & Boyle, 2005).

### Reporting summary

Further information on research design is available in the Nature Research Reporting Summary linked to this article.

## Supplementary information

Supplementary Information

Reporting Summary

## Data Availability

Data is available at 10.5281/zenodo.4419625.

## Code availability

Correlation analysis code is available at 10.5281/zenodo.4419625.
