# Supplementary material for: Trajectories of performance change indicate multiple dissociable links between working memory and fluid intelligence
Source: NPJ Sci Learn. 2021 Nov 29;6:33. doi: 10.1038/s41539-021-00111-w (PMC8630055; doi:10.1038/s41539-021-00111-w)
Supplement: Supplementary file 1 — Supplementary Information [file 41539_2021_111_MOESM1_ESM.pdf]

Cochrane & Green – Trajectories of performance change indicate multiple dissociable links between working memory and fluid intelligence

Supplementary Information

Supplementary Note 1: Regression using all three components of WM

In the primary text, regressions are only reported for components of WM change that had shown bivariate correlations with Gf. However, if all three components of WM are included as predictors in a single model, the same pattern emerges. Starting accuracy was not predictive of Gf when controlling for the other two components (*b*=0.117, CI=[-0.182,0.415], ΔR^2=0.006). In contrast, both other components of WM change were reliably predictive of Gf when controlling for other effects (rate of change *b*=-0.104, CI=[-0.205,-0.002], ΔR^2=0.042; ending accuracy *b*=0.402, CI=[0.055,0.749], ΔR^2=0.054). These same patterns held when using bootstrapped robust regression models as well (i.e., bootstrap 95% CI of robust regressions indicated the same patterns of reliability as the more typical regression reported above).

Supplementary Note 2: By-trial correlations

One additional method of assessing time-dependent changes in the correlations between WM and Gf is to calculate each WM trial's correlation with participants' Gf scores. Using this method we observed a large amount of variation in trial-wise correlations, however, there wass a clear increase over time (see Supplementary Figure 4). The R package **TEfits**[1] was used to fit an exponential function of change to the trial-wise correlations; the starting correlation was $r = .079$ while the asymptotic correlation was $r = .152$. In addition, across fits to 1,000 bootstrapped samples (i.e., resampling with replacement), 93.9% of resampled fits involved increases in trial-wise correlations with increasing trial number. Given the bootstrapped by-trial estimates of standard deviations of correlations, the correlation became higher than the first-trial correlation (with Cohen's d > 1) by the 12th trial. This further supports the argument that early WM task performance is less related to Gf (and thus further highlights potential issues with exceptionally short tasks being utilized as part of task batteries).

Supplementary Note 3: Nonlinear model formula (brms package)

```
clickAccuracy ~ inv_logit(thAsym) + (inv_logit(thStart) - inv_logit(thAsym)) * (2^((1 - trialNum)/(2 + 2^thRate)));
thAsym ~ feedback + taskOrder + (setSize | subjectID);
thStart ~ feedback + taskOrder + (setSize | subjectID);
thRate ~ feedback + taskOrder + (1 | subjectID);
```

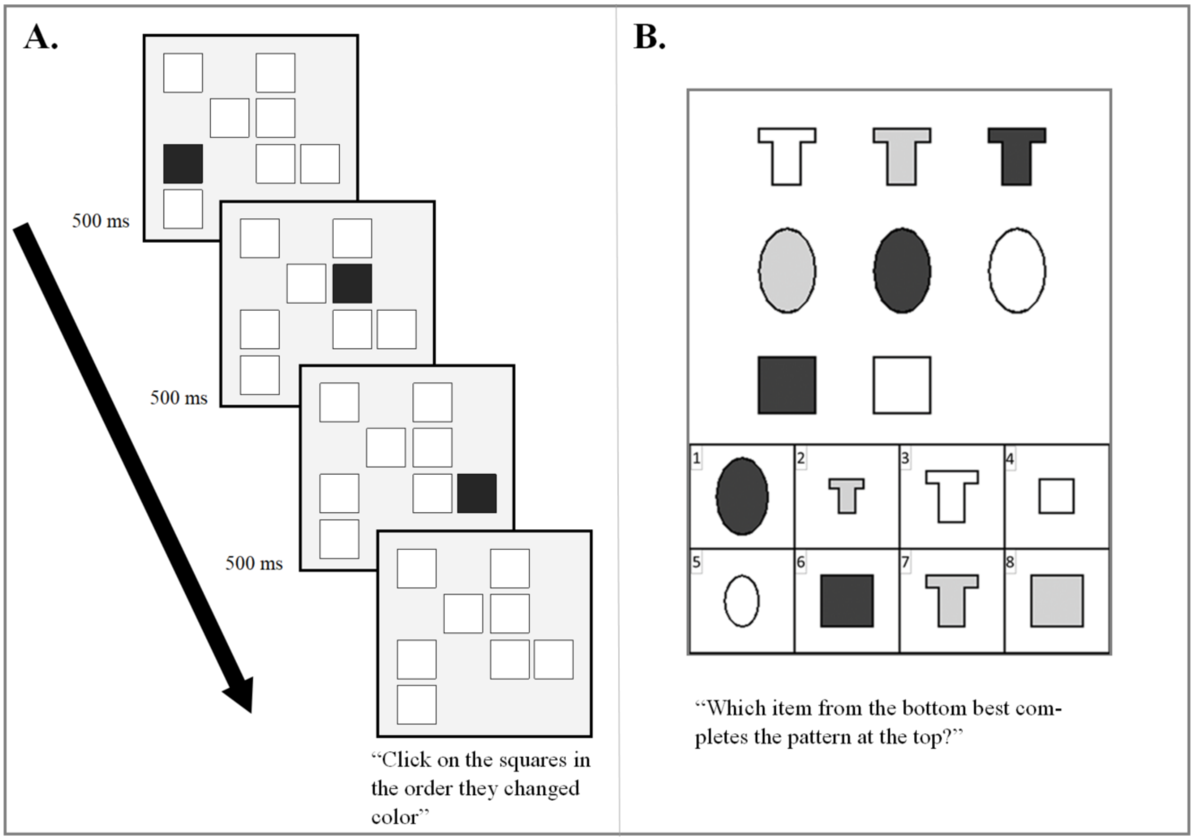

Supplementary Figure 1. Depictions of each task. (A) Spatial span example trial of set size 3. Boxes filled in one at a time, then participants were asked to repeat the sequence by clicking the boxes. (B) Matrix reasoning trial (10). The UCMRT involved the same task demands with superficially different displays.

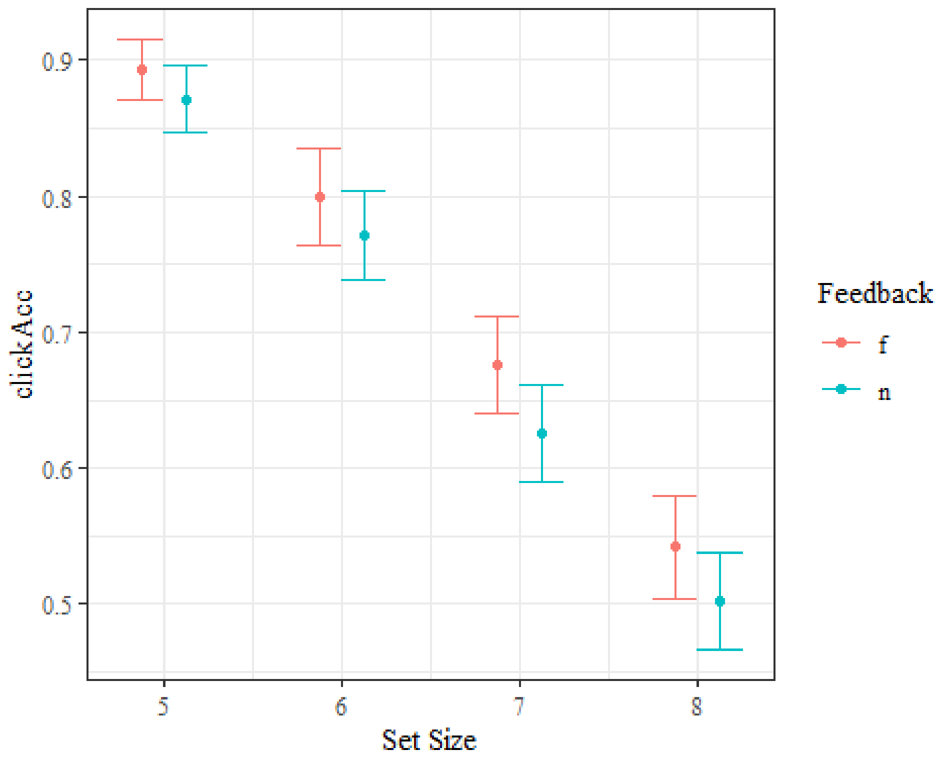

Supplementary Figure 2. Within-set-size average accuracy across all trials and participants within each feedback condition. The shape of this plot provides some justification for the use of the inverse logit transformation of accuracy utilized in model fitting (see formula above and Supplementary Figure 3). A wide range of accuracies were captured, indicating the ability to detect inter-participant differences in ability. Feedback was weakly associated with better overall performance ("f" indicates feedback-present, "n" indicates feedback-absent).

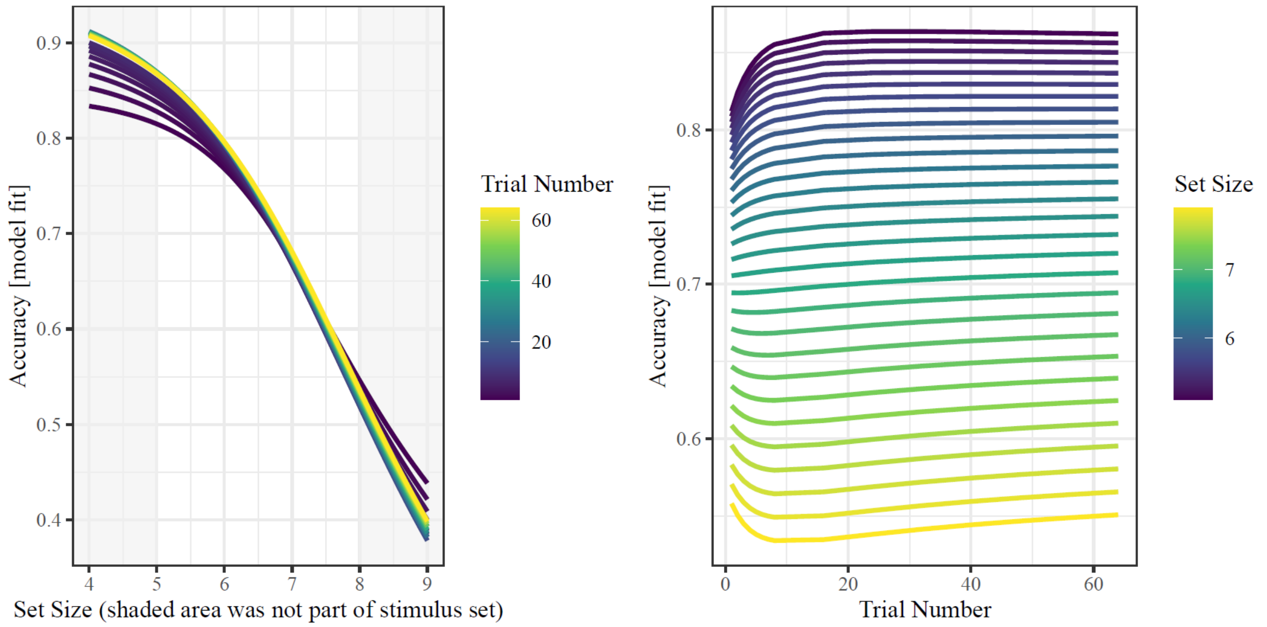

Supplementary Figure 3. Time-dependent changes in the psychometric function (i.e., accuracy varying by set size). Averages across the entire sample are displayed; single-participant estimates tended to demonstrate more change over time.

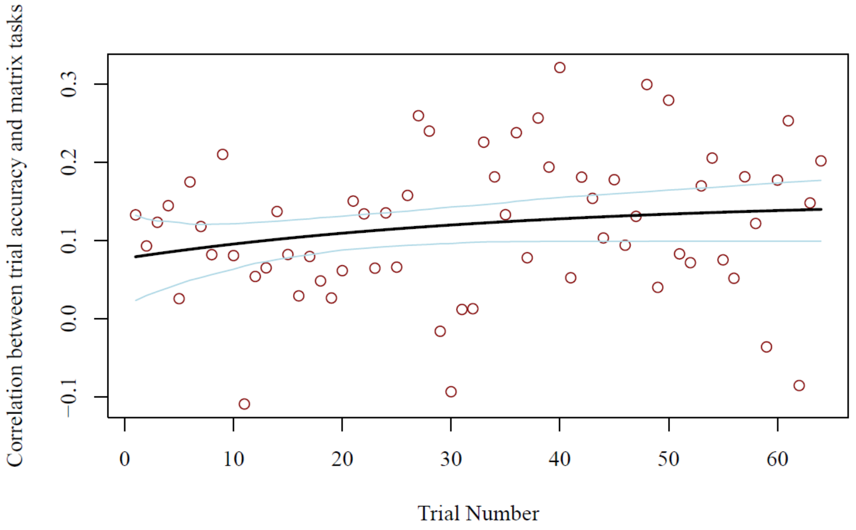

Supplementary Figure 4. Correlations between Gf and each trial of spatial span. Correlations increase with increasing trial number. Circles indicate each WM trial's Pearson correlation coefficient with Gf. Black line indicates overall fit to the data (exponential regression using ***TEfits****). Blue lines indicate 95% CI of estimated correlation coefficients.*

	Estimate	Est.Error	l-95% CI	u-95% CI	Rhat
thAsym_Intercept	1.38	0.17	1.07	1.73	1.01
thAsym_feedback	-0.22	0.23	-0.67	0.24	1.00
thAsym_taskOrder	0.02	0.23	-0.42	0.52	1.00
thStart_Intercept	1.21	0.17	0.90	1.57	1.00
thStart_feedback	-0.19	0.22	-0.62	0.23	1.00
thStart_taskOrder	-0.33	0.23	-0.79	0.13	1.00
thRate_Intercept	2.53	1.54	-0.65	5.35	1.02
thRate_feedback	1.41	1.54	-1.64	4.44	1.00
thRate_taskOrder	2.22	1.60	-1.03	5.30	1.00

Supplementary Table 1. Model output of fixed effects from nonlinear multi-level regression using ***brms***[2] *in R. Estimate provides point estimates, and Est.Error provides a measure of variability around the point estimates. The feedback-present condition was the reference group for the binary feedback variable, while the order variable was zero-centered (i.e., [-05., 0.5]). l-95% CI and u-95% CI denote the interval of likely values, and if 0 is outside this interval a parameter is considered reliable. Rhat indicates convergence if the value is near 1, with values below 1.05 conventionally being considered acceptable. Note that no effects of feedback or task order indicated reliability.*

Set Size	Time	Correlation
5	Beginning	r(85) = 0.19 [-0.02,0.38], BF_{log3} = 0.08
6	Beginning	r(85) = 0.2 [-0.01,0.39], BF_{log3} = 0.18
7	Beginning	r(85) = 0.17 [-0.05,0.36], BF_{log3}= -0.25
8	Beginning	r(85) = 0.08 [-0.12,0.31], BF_{log3} = -1.03

5	End	r(85) = 0.33 [0.13,0.52], BF_{log3} = 3
6	End	r(85) = 0.34 [0.13,0.53], BF_{log3} = 3.21
7	End	r(85) = 0.32 [0.13,0.5], BF_{log3} = 2.57
8	End	r(85) = 0.25 [0.05,0.42], BF_{log3} = 1.01

Supplementary Table 2. Correlations between Gf and WM accuracy, with model fits evaluated at each set size and by timepoint (i.e., beginning vs. end; trial 1 vs. trial 68).

Supplementary References

1. Cochrane, A. TEfits: Nonlinear regression for time-evolving indices. *J. Open Source Softw.* **5**, 2535 (2020).
2. Bürkner, P.-C. **brms** : An *R* Package for Bayesian Multilevel Models Using *Stan. J. Stat. Softw.* **80**, (2017).
