# Supplementary material for: Trajectories of performance change indicate multiple dissociable links between working memory and fluid intelligence
Source: NPJ Sci Learn. 2021 Nov 29;6:33. doi: 10.1038/s41539-021-00111-w (PMC8630055; doi:10.1038/s41539-021-00111-w)
Supplement: Supplementary file 2 — Reporting Summary [file 41539_2021_111_MOESM2_ESM.pdf]

nature portfolio

Corresponding author(s): Aaron Cochrane, PhD
University of Geneva
40 Boulevard du Pont-d'Arve
1211 Genève, Switzerland

Last updated by author(s): Oct 28, 2021

Reporting Summary

Nature Portfolio wishes to improve the reproducibility of the work that we publish. This form provides structure for consistency and transparency in reporting. For further information on Nature Portfolio policies, see our Editorial Policies and the Editorial Policy Checklist.

Statistics

For all statistical analyses, confirm that the following items are present in the figure legend, table legend, main text, or Methods section.

n/a	Confirmed	
☐	☒	The exact sample size (*n*) for each experimental group/condition, given as a discrete number and unit of measurement
☐	☒	A statement on whether measurements were taken from distinct samples or whether the same sample was measured repeatedly
☐	☒	The statistical test(s) used AND whether they are one- or two-sided<br>*Only common tests should be described solely by name; describe more complex techniques in the Methods section.*
☐	☒	A description of all covariates tested
☐	☒	A description of any assumptions or corrections, such as tests of normality and adjustment for multiple comparisons
☐	☒	A full description of the statistical parameters including central tendency (e.g. means) or other basic estimates (e.g. regression coefficient) AND variation (e.g. standard deviation) or associated estimates of uncertainty (e.g. confidence intervals)
☐	☒	For null hypothesis testing, the test statistic (e.g. *F*, *t*, *r*) with confidence intervals, effect sizes, degrees of freedom and *P* value noted<br>*Give P values as exact values whenever suitable.*
☐	☒	For Bayesian analysis, information on the choice of priors and Markov chain Monte Carlo settings
☐	☒	For hierarchical and complex designs, identification of the appropriate level for tests and full reporting of outcomes
☐	☒	Estimates of effect sizes (e.g. Cohen's *d*, Pearson's *r*), indicating how they were calculated

Our web collection on statistics for biologists contains articles on many of the points above.

Software and code

Policy information about availability of computer code

Data collection	Data was collected using Qualtrics in a Chrome browser.
Data analysis	Data used R; models and code are described in the Supplemental Information, and a repository for custom code is linked to in the manuscript.

For manuscripts utilizing custom algorithms or software that are central to the research but not yet described in published literature, software must be made available to editors and reviewers. We strongly encourage code deposition in a community repository (e.g. GitHub). See the Nature Portfolio guidelines for submitting code & software for further information.

Data

Policy information about availability of data

All manuscripts must include a data availability statement. This statement should provide the following information, where applicable:

- Accession codes, unique identifiers, or web links for publicly available datasets
- A description of any restrictions on data availability
- For clinical datasets or third party data, please ensure that the statement adheres to our policy

Data, and correlation analysis code, are available at http://doi.org/10.5281/zenodo.4419625.

Field-specific reporting

Please select the one below that is the best fit for your research. If you are not sure, read the appropriate sections before making your selection.

☐ Life sciences ☒ Behavioural & social sciences ☐ Ecological, evolutionary & environmental sciences

For a reference copy of the document with all sections, see nature.com/documents/nr-reporting-summary-flat.pdf

Behavioural & social sciences study design

All studies must disclose on these points even when the disclosure is negative.

Study description	The study is quantitative and cross-sectional.
Research sample	The sample was 87 undergraduate students at the University of Wisconsin-Madison. All were young adults, and 50 were female. This sample was representative of the particular undergraduate population but not representative of the broader world; see the Methods for a discussion of sample choice and generalizability.
Sampling strategy	Samples were recruited through an automated system for participating for extra credit. A heuristic target sample of 100 was chosen due to the more-than-sufficient power it would provide (given previous results; see Methods).
Data collection	The experimenter, who was blind to the hypotheses of the study, initiated the computerized tasks on Dell desktop computers running Windows 7. Data collection used a standard PC mouse. The experimenter was also blind to the presence or absence of feedback to the participant. All instructions were provided in writing. No other persons were in the room as the participant completed the tasks.
Timing	Start date: 12 March 2019; Stop date: 15 November 2019
Data exclusions	Participants' data were excluded if they met any one of three pre-established criteria. First, we included very easy "catch" trials in our working memory task, and participants had to achieve at least 50% correct on these trials to be excluded. Second and third, participants' accuracy on each of our matrix reasoning tasks was compared to chance (using a one-tailed binomial test). These criteria were pre-established and informed our task design. The numbers of excluded participants (21) is reported in the manuscript.
Non-participation	No participants dropped out or chose not to participate at any point.
Randomization	Participants were pseudo-randomly assigned to experimental groups manipulating accuracy feedback and counterbalancing task orders. A spreadsheet of group assignments was created prior to running participants, and each participant was assigned to their group in the order that the participant participated.

Reporting for specific materials, systems and methods

We require information from authors about some types of materials, experimental systems and methods used in many studies. Here, indicate whether each material, system or method listed is relevant to your study. If you are not sure if a list item applies to your research, read the appropriate section before selecting a response.

Materials & experimental systems

n/a	Involved in the study	
☒	☐	Antibodies
☒	☐	Eukaryotic cell lines
☒	☐	Palaeontology and archaeology
☒	☐	Animals and other organisms
☐	☒	Human research participants
☒	☐	Clinical data
☒	☐	Dual use research of concern

Methods

n/a	Involved in the study	
☒	☐	ChIP-seq
☒	☐	Flow cytometry
☒	☐	MRI-based neuroimaging

Human research participants

Policy information about studies involving human research participants

Population characteristics	See above
Recruitment	Participants were recruited through the SONA online software, and signed up for 1-hour study time spots without having any prior knowledge of the study content.
Ethics oversight	University of Wisconsin - Madison: Education and Social/Behavioral Science Institutional Review Board

Note that full information on the approval of the study protocol must also be provided in the manuscript.
